# The identity of the discriminator base has an impact on CCA addition

**DOI:** 10.1093/nar/gkv471

**Published:** 2015-05-09

**Authors:** Sandra Wende, Sonja Bonin, Oskar Götze, Heike Betat, Mario Mörl

**Affiliations:** Institute for Biochemistry, University of Leipzig, Brüderstrasse 34, 04103 Leipzig, Germany

## Abstract

CCA-adding enzymes synthesize and maintain the C-C-A sequence at the tRNA 3′-end, generating the attachment site for amino acids. While tRNAs are the most prominent substrates for this polymerase, CCA additions on non-tRNA transcripts are described as well. To identify general features for substrate requirement, a pool of randomized transcripts was incubated with the human CCA-adding enzyme. Most of the RNAs accepted for CCA addition carry an acceptor stem-like terminal structure, consistent with tRNA as the main substrate group for this enzyme. While these RNAs show no sequence conservation, the position upstream of the CCA end was in most cases represented by an adenosine residue. In tRNA, this position is described as discriminator base, an important identity element for correct aminoacylation. Mutational analysis of the impact of the discriminator identity on CCA addition revealed that purine bases (with a preference for adenosine) are strongly favoured over pyrimidines. Furthermore, depending on the tRNA context, a cytosine discriminator can cause a dramatic number of misincorporations during CCA addition. The data correlate with a high frequency of adenosine residues at the discriminator position observed *in vivo*. Originally identified as a prominent identity element for aminoacylation, this position represents a likewise important element for efficient and accurate CCA addition.

## INTRODUCTION

As translational adapter molecules, tRNAs deliver amino acids to the nascent polypeptide during protein synthesis (1). Synthesized as precursor molecules, these transcripts have to undergo a series of processing and modification steps, converting them into mature and functional tRNAs. The maturation process includes removal of 5′-leader and 3′-trailer sequences, splicing events, base modifications as well as the addition of the 3′-terminal CCA sequence (2,3). In these processing steps, tRNAs have to be recognized by a variety of different maturation enzymes (3,4). One of these enzymes is tRNA nucleotidyltransferase (CCA-adding enzyme), which is responsible for the incorporation of the 3′-terminal CCA triplet, generating the site of aminoacylation (5–7). According to their structural organization, CCA-adding enzymes are divided into class I (archaeal type) and class II (bacterial/eukaryotic type) enzymes (7,8). These enzymes follow different strategies to incorporate the individual nucleotides in a highly accurate process. Class I enzymes use a combination of amino acids in the nucleotide binding pocket and sugar-phosphate backbone positions of the tRNA to selectively bind CTP and ATP, while class II enzymes rely solely on the base-specific hydrogen bonding properties of a set of amino acid residues in the binding pocket (9). To interact with a tRNA primer, however, both types of enzymes use a similar strategy, depending on size, shape and charge complementarity (7,9). These interactions are predominantly formed with the sugar-phosphate backbone of the top-half of a tRNA molecule (7,9–11). This allows sequence-independent substrate recognition, leading to efficient CCA addition on all tRNAs within a cell. As such 3′-terminal stems are also found in non-tRNA-like transcripts, several further RNAs are described as alternative substrates for CCA addition in tobacco mosaic virus (12), maize mitochondria (13) and chloroplasts of tobacco (14). Furthermore, the human spliceosomal U2 snRNA (15) as well as the eukaryotic mascRNA (16) carry non-encoded CCA sequences at the 3′-terminus. Interestingly, CCA-carrying non-tRNA substrates in maize do not fold into a 3′-terminal hairpin element, indicating that a tRNA-like 3′-end is not an absolute prerequisite for being accepted as a substrate for CCA addition (13). Rather, it seems that the RNA sequence in itself might have an impact on the substrate acceptance of CCA-adding enzymes.

In the present study, we analysed substrate requirements of the human CCA-adding enzyme. The data confirm that transcripts lacking a tRNA-like 3′-end are also accepted for CCA addition. Most surprising, however, is the observation that the identity of the 3′-terminal nucleotide, corresponding to the discriminator position in tRNAs, has a great impact on the efficiency of CCA incorporation. Originally described as an identity element for tRNA recognition by cognate aminoacyl-tRNA synthetases (17,18), the discriminator base represents a likewise important substrate recognition element for tRNA nucleotidyltransferases.

## MATERIALS AND METHODS

### Preparation of RNA substrates

For the generation of a randomized pool of RNA molecules, a synthetic DNA pool consisting of 73 randomized positions was used (Purimex). At the 5′-end, the pool carried 15 nucleotides of the 3′-part of the T7 promoter sequence, followed by two G residues for efficient transcription. The 3′-end consisted of the first 15 nucleotides of the Hepatitis delta virus ribozyme (HDV). T7 promoter and HDV sequence were completed by overlap extension polymerase chain reaction (PCR). The PCR product was transcribed in the presence or absence of α-^32^P-ATP. Homogeneous transcript 3′-ends were generated by HDV ribozyme cleavage, and the resulting 2′,3′-cyclic phosphate was removed by T4 polynucleotide kinase (19).

### Recombinant protein expression and purification

Recombinant CCA-adding enzymes were prepared as described (20,21).

### *In vitro* nucleotide incorporation

Fifteen picomol RNA pool (spiked with 2.5 pmol radioactively labelled pool) was incubated for 2 h with 200 ng human CCA-adding enzyme as described (22). For time course analysis and testing of individual substrate candidates, 5 pmol of radioactively labelled RNA was incubated with 50–100 ng enzyme for 30 min to 2 h. In the competition study, 2.5 pmol of each tRNA was mixed and incubated with 100 ng human CCA-adding enzyme in a final volume of 20 μl for various time points (21). Reaction products were ethanol precipitated, size separated by denaturing polyacrylamide gel electrophoresis and visualized by autoradiography.

### Kinetic analysis of CCA addition

For steady-state Michaelis–Menten kinetics, 15–200 ng enzyme were incubated with RNA transcript titrated between 1 and 10 μM according to Wolf *et al*. (23). Kinetic parameters of three to five independent experiments were analysed using curve-fitting by non-linear regression (GraphPadPrism). As the transcripts are not soluble at excessive saturating conditions, the obtained kinetic parameters represent apparent values (24,25).

### Sequence analysis of reaction products

Reaction product bands were size-separated on a denaturing polyacrylamide gel, cut out and eluted from the gel matrix (21). RNA 3′-ends were ligated to a DNA oligonucleotide (Purimex) carrying one single RNA nucleotide (UMP) at the phosphorylated 5′-end, reverse transcribed and amplified (21). The resulting cDNA was subjected to a 5′ RACE procedure (Invitrogen). PCR products were cloned into pCR 2.1 Topo® and sequences of reaction products determined (21). Only full-length sequences were considered for further analysis.

### RNA secondary structure predictions

RNA secondary structures were predicted using the RNA Vienna package (RNAfold) (26). Structure presentations were done using VARNA (27). For tRNA structures, dot bracket annotation of the tRNA database was used (28).

## RESULTS

### *In vitro* selection of RNA substrates for CCA addition

**Figure 1.**
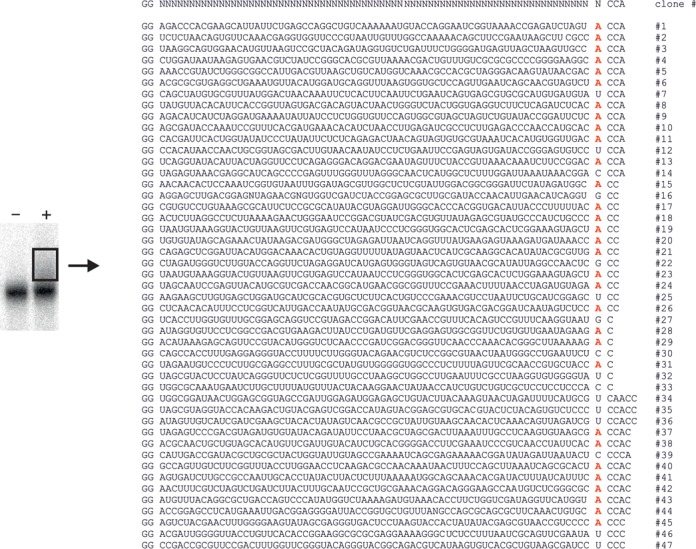
Substrates for CCA addition. Left: Randomized RNA sequences were incubated in presence (+) and absence (-) of the human CCA-adding enzyme. The enzyme incorporates nucleotides in some transcripts, leading to a reduced electrophoretic mobility, visible as a smear (boxed) above the main band of the RNA pool. Right: Sequences of 47 individual RNA substrates retrieved from the shifted transcripts. Due to the construction of the library for efficient T7 transcription, all sequences start with GG. Nucleotides incorporated by the CCA-adding enzyme are shown on the right. The base located immediately upstream of the added nucleotides (corresponding to the tRNA discriminator position 73) is shown separately. Here, a strong excess of adenosine (red) is visible. While most of the clones carry a complete or partial CCA end, some show additional C residues incorporated.

As several of the described additional CCA-carrying RNAs do not fold into a structure corresponding to the top half of a tRNA, the general substrate requirement for the human CCA-adding enzyme was investigated. A pool of randomized RNA sequences (5.4 × 10^13^ molecules) of approximate tRNA length was synthesized as radioactively labelled transcripts with homogeneous 3′-ends using T7 RNA polymerase and HDV ribozyme (19). For efficient transcription, the corresponding DNA template carried two G residues at the transcription start site, leading to a total RNA length of 75 nucleotides. The transcripts were incubated with recombinant human CCA-adding enzyme in the presence of nucleotides. Reaction products were separated on a denaturing polyacrylamide gel and visualized by autoradiography. While the incubation without enzyme gave rise to a relatively sharp single band, the activity of the enzyme led to a shifted region of smear above the substrate band, indicating nucleotide addition on a subset of transcripts (Figure 1). The fact that no sharp bands are visible is caused by the complexity of the RNA molecules within the pool as well as the varying number of nucleotides added.

**Figure 2.**
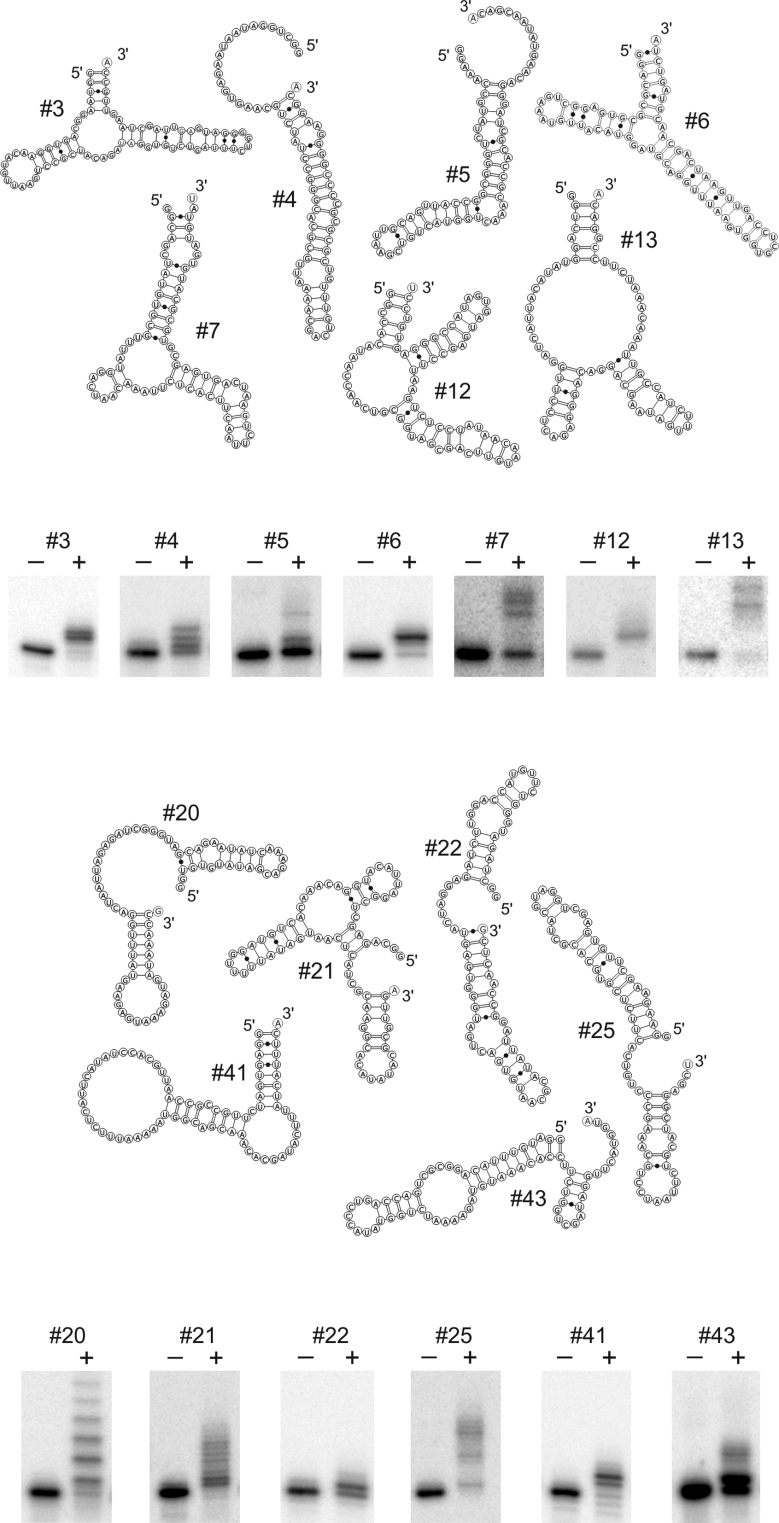
Individual candidate transcripts are true substrates for CCA addition. From the list of candidates presented in Figure 1, 13 RNA molecules were tested individually with the CCA-adding enzyme (+). The negative control represents the transcripts incubated without enzyme. All candidates show nucleotide additions with varying efficiency, ranging from the incorporation of a single nucleotide (partial CCA end, #22) to the addition of three or more residues. The secondary structure models show that most of the candidates carry base-paired 5′- and 3′-ends with 3′-terminal nucleotide overhangs, corresponding to an acceptor stem-like structure. Yet, also single-stranded 3′-ends are tolerated for nucleotide addition, although at a rather low efficiency (#5, #25, #43).

Reaction products were isolated and subjected to 3′- and 5′-RACE analysis. Sequence determination of individual clones revealed 47 distinct full-length RNA molecules carrying additional C and A residues incorporated at the 3′-terminus (Figure 1). To confirm that these transcripts indeed represent true substrates for CCA addition, 13 arbitrarily chosen candidates were cloned without CCA terminus and the corresponding radioactively labelled *in vitro* transcripts were tested individually for CCA addition (Figure 2). All of the transcripts showed a reduced electrophoretic mobility in the gel, indicating that these RNAs were accepted for nucleotide incorporation by the enzyme and that presumably all of the candidates listed in Figure 1 represent substrates for CCA addition. Interestingly, structure predictions suggest that several of the candidates (#5, #25 and #43) carry a single-stranded 3′-end, a further indication that a 3′-terminal hairpin is not an absolute prerequisite for CCA addition, as observed for mitochondrial mRNAs in maize (13). For most of the substrates, however, base-paired 5′- and 3′-ends with some similarity to a tRNA acceptor stem are predicted (Figure 2).

**Figure 3.**
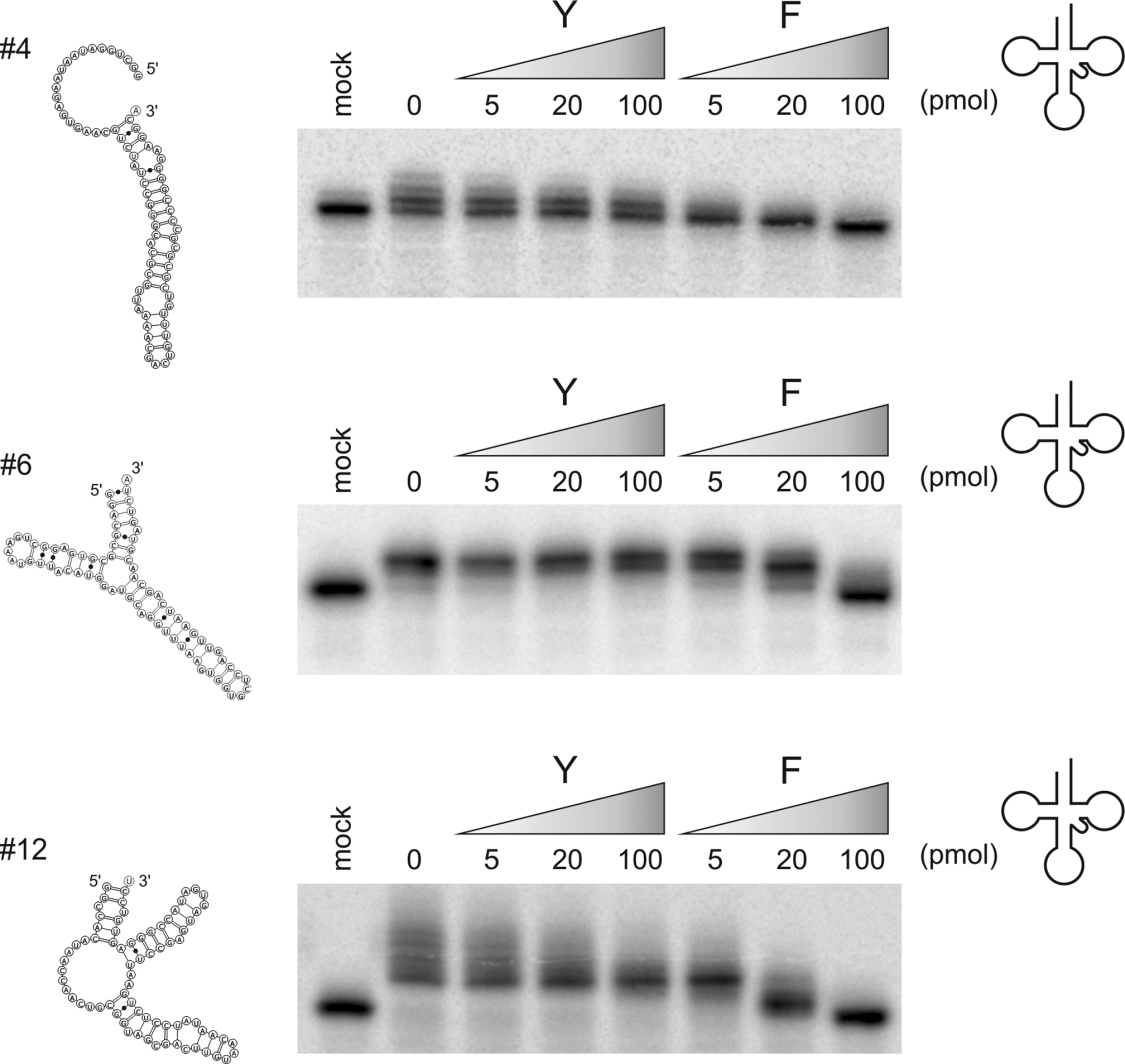
Competition studies with candidates and tRNAs. Substrate candidates #4, #6 and #12 were incubated as radioactively labelled transcripts with increasing concentrations of unlabelled tRNA as competitor. While the yeast tRNA^Phe^ (F) shows an efficient competition leading to reduced NTP incorporation into the candidates, the human mitochondrial tRNA^Tyr^ (Y) is competing less efficiently. The nucleotide addition in the presence of intact tRNA indicates that the candidates are indeed competent substrates, although at a lower efficiency compared to tRNA^Phe^.

To investigate the efficiency of CCA addition on these transcripts, steady-state kinetic parameters for several individual candidates were determined. As substrates for Michaelis–Menten kinetics, candidates #4, #6 and #12 were chosen arbitrarily. All resulting apparent *K_M_* values lie in a range between 1.4 and 7.3 μM (Table 1), similar to those obtained for tRNAs (Table 2), while the turnover number *k*_cat_ was considerably reduced. Hence, these data indicate that the selected candidate RNAs represent substrates for CCA addition. Yet, it is possible that in a competitive situation, the enzyme still prefers tRNAs over the artificial substrates. To investigate the substrate performance of candidates #4, #6 and #12 in the presence of tRNA substrates, CCA addition on radioactively labelled transcripts was monitored in the presence of an increasing amount of unlabelled tRNA as a competitor (Figure 3). As the substrate efficiency can vary dramatically from tRNA to tRNA, two different tRNAs were selected. The yeast tRNA^Phe^ represents one of the best studied substrates for CCA addition and the *in vitro* transcript folds into a structure very similar to that of the native tRNA (24,29–31). Furthermore, the human mitochondrial tRNA^Tyr^ was selected, representing a natural substrate for the human CCA-adding enzyme. In addition, this transcript is also frequently used for *in vitro* CCA addition (21,32). For candidate #4, tRNA^Tyr^ is a rather weak competitor and only higher ratios (4:1, 20:1) show a detectable reduction in nucleotide incorporation in this candidate (Figure 3, upper panel, left). In contrast, the presence of tRNA^Phe^ immediately leads to a dramatic reduction in nucleotide addition on this transcript (Figure 3, upper panel, right). Similar results were obtained for candidates #6 and #12 (Figure 3, central and lower panel), indicating that a perfectly structured tRNA^Phe^ is a good competitor for CCA addition, while tRNA^Tyr^, showing the typical structural features of mitochondrial tRNAs (33), is less efficient.

**Table 1.** Kinetic parameters determined for three RNA candidates as substrates for the human CCA-adding enzyme

Substrate	*K_M_* (μM RNA)	*k*_cat_ (s^−1^)
#4	1.40 +/− 0.50	0.008 +/− 0.008
#6	5.30 +/− 2.60	0.046 +/− 0.011
#12	7.30 +/− 3.10	0.004 +/− 0.001

**Table 2.** Kinetic parameters determined for the human CCA-adding enzyme and tRNA substrates with different discriminator bases

Substrate	*K_M_* (μM tRNA)	*k*_cat_ (s^−1^)
tRNA^Tyr^-A (wt)	3.3 +/− 1.2	0.18 +/− 0.03
tRNA^Tyr^-U	1.9 +/− 1.4	0.06 +/− 0.01
tRNA^Pro^-C (wt)	1.1 +/− 0.4	0.13 +/− 0.01
tRNA^Pro^-A	3.8 +/−1.6	0.33 +/− 0.06

While the kinetics as well as the competition data clearly show that the selected candidates are readily accepted for CCA addition, the most surprising result is that 31 out of 47 candidates (65.9%) carry an adenosine at the 3′-terminal base position, immediately upstream of the added CCA end (Figure 1, Supplementary Table S1). In tRNAs, this position corresponds to the discriminator base, an important identity element for aminoacyl-tRNA synthetases (17,18,34–36). As most of the synthetases prefer an adenosine residue at this position, 62.2% of the tRNAs in all three kingdoms carry this base at the corresponding position (37). Interestingly, this base distribution is very similar to the observed base frequencies at the 3′-end of the selected RNA candidates shown in Figure 1 (Supplementary Table S1). While tRNAs show a strong preference for purines (85.1%) over pyrimidines (14.9%) (37), the candidate transcripts for CCA addition show a similar overrepresentation of G and A residues (72.3%) compared to pyrimidines (27.6%). The prevalence of adenosine at the 3′-ends of the substrate RNAs is not the result of a base preference for HDV ribozyme cleavage during substrate preparation, as it was demonstrated that this ribozyme has no preference for nucleotides located immediately upstream of the cleavage position (19). Furthermore, 3′-end analysis of the original pool showed that only 20 out of 75 sequences (27%) carried an A residue at the 3′-terminus, while G, T and C were found in 13/75 (17%), 25/75 (33%) and 17/75 (17%), respectively. These numbers indicate that the RNA pool did not contain a bias for 3′-terminal purines or especially adenosine. Rather, pyrimidines, and especially U, are slightly overrepresented. Furthermore, to rule out the possibility that the CCA-adding enzyme itself is able to add this adenosine position before synthesizing the CCA terminus, substrate candidate #3 was synthesized without the 3′-terminal A residue and incubated with the CCA-adding enzyme under standard nucleotide incorporation conditions (Supplementary Figure S2). No nucleotide incorporation was visible, showing that the CCA-adding enzyme is not able to restore this nucleotide, consistent with earlier findings on 3′-terminally truncated tRNA substrates (38). Hence, the high frequency of adenosine residues upstream of the CCA-terminus of the selected candidate RNAs must originate from the preference of the CCA-adding enzyme for substrates ending with this base.

### The CCA-adding enzyme shows a preference for tRNAs with a purine discriminator

**Figure 4.**
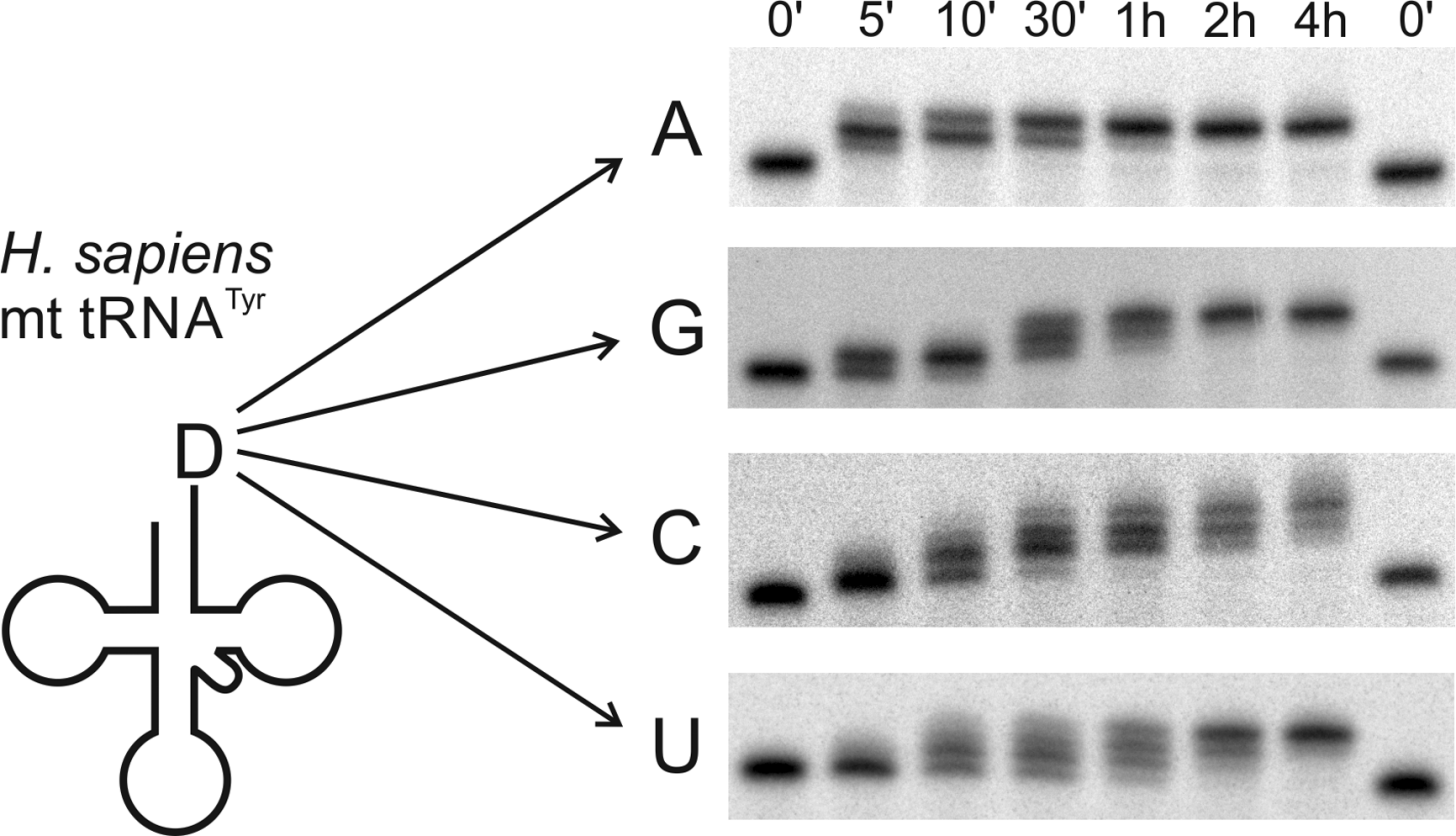
The discriminator base affects CCA addition. From 5 min to 4 h of incubation with human CCA-adding enzyme, the incorporation of CCA termini in the human mitochondrial tRNA^Tyr^ was monitored by the appearance of shifted bands in the polyacrylamide gels. The wild-type transcript with A73 shows the fastest addition, with a complete turnover after 1 h. Transcripts with G73 or U73 show a complete CCA addition after 2 or 4 h, respectively. While the reaction stops after addition of three residues to these substrates, the tRNA ending with C73 shows further band shifts, indicating the incorporation of additional nucleotides.

As the high prevalence of adenosine residues at the 3′-end of the substrate RNAs indicates a possible impact of this residue on CCA addition, we investigated whether the corresponding discriminator position in a tRNA (position 73 according to Sprinzl et al. (39)) is not only an identity element for aminoacylation, but also affects CCA addition. Variants of the human mitochondrial tRNA^Tyr^ with discriminator positions A (corresponding to the wild-type situation), G, U and C were prepared by mutagenesis and subsequent *in vitro* transcription of the corresponding DNA constructs. The resulting radioactively labelled transcripts were individually tested for CCA incorporation in a time series (Figure 4). The resulting band patterns on the polyacrylamide gels show that all four tRNA variants were accepted by the human CCA-adding enzyme and were elongated for up to three nucleotides, corresponding to the incorporation of complete or partial CCA ends. As the time courses indicate, the different discriminator positions have a strong impact on efficiency and speed of CCA incorporation. For tRNA^Tyr^ versions with A73 or G73, complete substrate turnover is visible after 1 to 2 h (complete shift of the resulting product bands), while the same tRNA with U73 or C73 shows a completed reaction only after 4 h. Similar results were obtained for the enzymes of *Escherichia coli* and *Archaeoglobus fulgidus* (Supplementary Figure S3).

While tRNA versions with A, G or U at position 73 show precise addition of three residues, the product band of tRNA^Tyr^ with C73 shows additional migration shifts, revealing the incorporation of extra nucleotides (Figure 4). Furthermore, the initial addition of the first nucleotides of the CCA terminus is also much faster for tRNA^Tyr^ with A73 or G73, represented by the corresponding band shifts after 5 min. Transcripts with C73 show only a weak nucleotide addition at this time point, while tRNA^Tyr^ with U73 has the slowest incorporation rate, and almost no nucleotide addition is visible after 5 min of incubation. Accordingly, the faster CCA addition observed for tRNA^Tyr^ with A73 is consistent with the data obtained from the selection assay, indicating that the discriminator position indeed plays an important role for an efficient addition of the CCA terminus.

**Figure 5.**
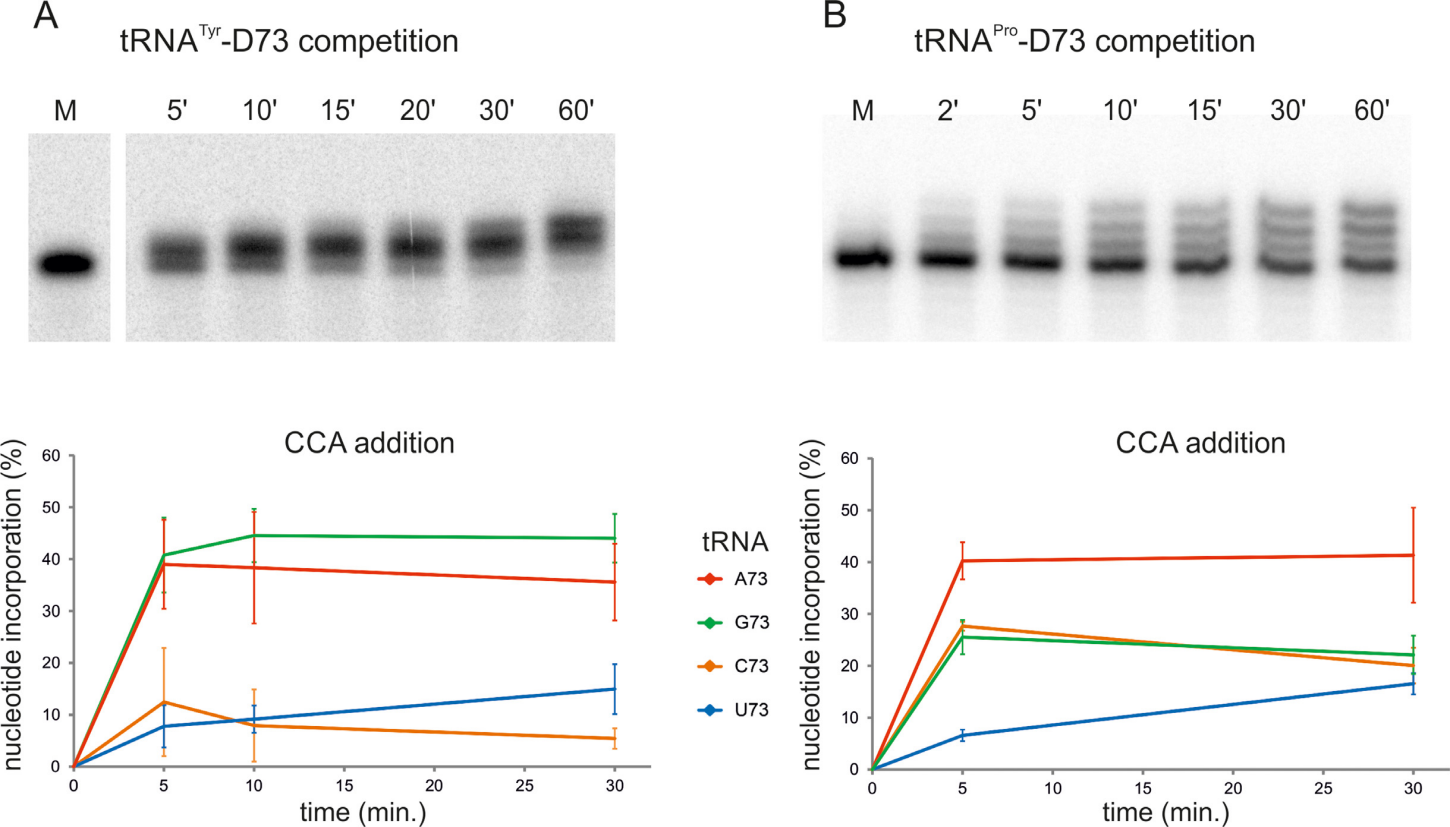
Competition study with tRNA variants carrying different discriminator bases. (**A**) Equimolar amounts of radioactively labelled transcripts of human mitochondrial tRNA^Tyr^ with A73, G73, C73 and U73 were incubated with the human CCA-adding enzyme in a time series. At the indicated time points, reaction products were separated by denaturing gel electrophoresis. The reduced migration of the signal bands indicates the addition of one to three nucleotides, corresponding to the CCA terminus. Lower panel: quantitative analysis of individual nucleotide additions. After the first time point, a clear preference for tRNA carrying purine discriminators is detectable. About 40% of the analysed clones carry either G or A at position 73. Over the whole time course, tRNAs with A73 or G73 represent the preferred substrates for CCA addition, while transcripts ending with C73 or U73 show a dramatically reduced CCA addition. (**B**) Competition experiment with human tRNA^Pro^. The experiment was conducted according to (A). tRNA^Pro^ with A73 is a much better substrate for CCA addition than the native transcript with C73. Yet, CCA incorporation in this transcript is as efficient as for tRNA^Pro^ with G73. While again a purine (adenosine) discriminator is highly preferred, the wild-type tRNA^Pro^ with a pyrimidine discriminator is a much better substrate than in the case of tRNA^Tyr^, indicating that the context of tRNA^Pro^ compensates for the non-optimal discriminator base.

For a more direct comparison of the impact of the discriminator base on CCA addition, a competition study was performed. Equimolar amounts of radioactively labelled human tRNA^Tyr^ transcripts with discriminator bases A, G, C and U were incubated with the human CCA-adding enzyme in the presence of nucleotides. After various time points ranging from 5 to 60 min, reaction products were separated on a denaturing polyacrylamide gel (Figure 5A). To quantify tRNA discriminator variants accepted as substrates for CCA addition, shifted bands from time points 5, 10 and 30 min were isolated and characterized by 3′-end sequencing. Reaction products after 60 min incubation were not taken into consideration, because the migration position indicates fully extended tRNAs carrying complete CCA ends. To compare efficiencies of CCA incorporation, such a saturation of the reaction has to be avoided. For each time point, three independent experiments were performed, resulting in 200–300 tRNA 3′-end sequences per indicated time point. In terms of discriminator identity, the relative abundance of tRNAs with complete or partial CCA ends was calculated.

Already after 5 min of incubation, the CCA-adding enzyme shows a strong preference for tRNAs carrying a purine base at the discriminator position, leading to 38 and 43% of CCA addition to tRNA with A73 or G73, respectively. Transcripts carrying a pyrimidine discriminator were extended only in 6 (U73) or 13% (C73). This tendency persisted for the whole incubation period up to 30 min. While after 5 and 10 min only partial CCA additions were detectable, complete CCA incorporations were observed exclusively at the last time point (30 min). This result indicates that the incubation conditions did not lead to any reaction saturation interfering with a quantitative analysis.

To obtain quantifiable data on the discriminator preference, steady-state kinetic analyses were conducted using tRNA^Tyr^ with an A residue at position 73 (showing efficient CCA addition) and a tRNA^Tyr^ version carrying U73 (showing inefficient CCA addition). For both transcripts, *K_M_* values (3.3 versus 1.9 μM) were obtained that were not significantly different (*P*-value: 0.3). *k*_cat_, however, dropped from 0.18 s^−1^ for tRNA^Tyr^-A73 to 0.06 s^−1^ for tRNA^Tyr^-U73, representing a significant 3-fold reduction in turn over (*P* = 0.006) (Table 2).

### CCA addition on tRNAs with cytidine as discriminator

**Figure 6.**
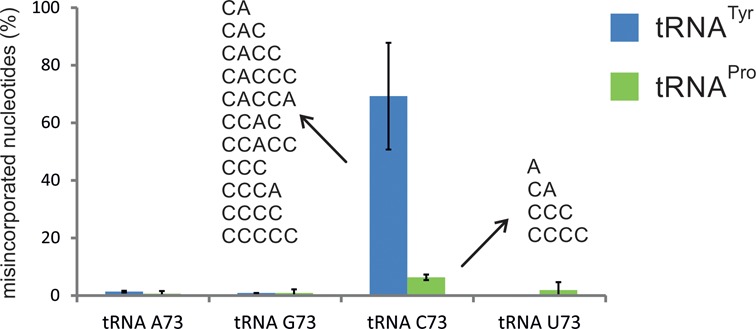
Erroneous nucleotide additions. tRNA substrates isolated in the competition experiment were analysed for nucleotide misincorporation catalysed by the CCA-adding enzyme. Whereas tRNA^Tyr^ (blue bars) with A, G or U residues at position 73 showed almost exclusively correct CCA additions, ∼70% of the corresponding transcripts with C73 carried misincorporations and extra nucleotides. Order and number of incorporated C and A residues showed a strong variability, leading to sequences with up to five added nucleotides in varying combinations of C and A. tRNA^Pro^ (green bars), on the other hand, showed only slightly increased misincorporation, if the discriminator base is a cytidine. Again, the structural context of this tRNA seems to compensate for the unfavourable C73 position.

Due to a highly selective nucleotide binding pocket, bacterial and eukaryotic CCA-adding enzymes exhibit an impressive specificity for the addition of CTP and ATP (40). Accordingly, exclusively C and A additions were observed in the competing tRNA candidates (Figure 5A). However, tRNA^Tyr^ with C73 represents a rather poor substrate, resulting in a low rate of nucleotide incorporations compared to tRNAs with purine discriminators. Yet, a great amount (70%) of tRNA-C73 substrates with nucleotide additions carried a dramatic number of misincorporations in the CCA sequence (Figure 6). The CCA-adding enzyme still incorporated C and A residues, but order and number of nucleotide additions deviated strongly from the expected CCA sequence. In addition, the enzyme added more than the expected nucleotide triplet, leading to tRNAs ending in a stretch of 4–5 incorporated residues like CACC(C) or CCCC(C). In contrast, similar misincorporations in tRNA-A73 (CCC) or tRNA-G73 (CCCA) were observed only in ∼1% of the analysed sequences, while tRNA-U73 did not show any erroneous nucleotide additions.

The C73-dependent nucleotide misincorporation might reflect the dramatic underrepresentation of cytosine discriminators in nature, where only 3.9% of all tRNAs carry this element (37). To test whether a naturally occurring C73 on a tRNA also leads to erroneous CCA addition, we analysed the discriminator impact on the human tRNA^Pro^ carrying a C73 position that is highly conserved in eukaryotes. Similar to tRNA^Tyr^, a competition experiment with tRNA^Pro^ transcripts with C, U, G and A as discriminator bases was performed (Figure 5B). Under non-saturating reaction conditions, 250–260 products per time points 5 and 30 min were investigated. Again, transcripts with A73 were highly preferred by the human CCA-adding enzyme, leading to a relative abundance of 40% among tRNAs with complete or partial CCA ends (Figure 5B). Transcripts with U73 were again the least efficient substrates (7% after 5 min). tRNA^Pro^ with C73, however, showed a strong discrepancy in comparison to tRNA^Tyr^, as this transcript was as efficient as tRNA^Pro^-G73 for CCA addition (28 versus 26% after 5 min). This tendency remained constant over the whole time period, indicating that a cytosine discriminator of tRNA^Pro^ is readily accepted by the CCA-adding enzyme, in contrast to tRNA^Tyr^. This is further corroborated by the fact that only 6% of tRNA^Pro^-C73 showed C and A misincorporations (Figure 6). Nevertheless, the kinetic parameters for tRNA^Pro^-C73 (wt) and tRNA^Pro^-A73 support an A73 preference also in this tRNA (Table 2). Although the *K_M_* values show a 3.5-fold difference (1.1 versus 3.8 μM), this difference is not significant (*P* = 0.05). *k*_cat_ shows a slightly better turnover for tRNA^Pro^-A73 (0.33 s^−1^ compared to 0.13 s^−1^ for tRNA^Pro^-C73). Here, the difference is highly significant (*P* = 0.005).

In addition, both tRNA^Pro^ versions were tested as substrates in the kinetic analysis of the class I CCA-adding enzyme of *A. fulgidus*, as this class of enzymes follows a different nucleotide selection mechanism compared to the human class II enzyme (see Introduction). As *A. fulgidus* represents a thermophilic Archaeon, the CCA-adding enzyme requires incubation at elevated temperatures (50°C). Hence, the structurally rather unstable tRNA^Tyr^ carrying five A–U pairs in the acceptor stem was not used for this analysis. The kinetic parameters for CCA addition on the structurally more stable tRNA^Pro^ are summarised in Supplementary Table S2. *k*_cat_ differs 2.4-fold between tRNA^Pro^-C73 (0.014 s-1) and tRNA^Pro^-A73 (0.033 s-1) with very high significance (*P* = 0.0001). *K_M_* shows a similar difference (1.5 μM for tRNA^Pro^-C73, 0.6 μM for tRNA^Pro^-A73), though at a somewhat lower statistical significance (*P* = 0.005).

## DISCUSSION

### The human CCA-adding enzyme shows a relaxed substrate specificity

Encoded by a single gene with a mitochondrial import sequence, the eukaryotic CCA-adding enzyme has to deal not only with canonical cytosolic tRNAs as substrates but also accepts structurally deviating mitochondrial counterparts (20,41). In nematodes and mites, this situation comes to an extreme, as many mitochondrial tRNAs show dramatic size reductions, leading to hairpin-like transcripts (42–45). Yet, these reduced tRNAs are bona fide substrates for CCA addition (45). Furthermore, non-tRNA-like substrates are described as well, ranging from viral, mitochondrial and chloroplast mRNAs to small noncoding transcripts like U2 snRNA or mascRNA (12,13,46,14–16). While most of these transcripts carry 3′-terminal hairpin structures resembling a tRNA acceptor stem, there is evidence that RNAs that do not fold into such a hairpin element are also substrates for CCA addition, as several microRNAs were shown to carry non-encoded CCA-termini (47). Our data indicate that the CCA-adding enzyme of *Homo sapiens* also tolerates a great variety of different RNA structures for nucleotide incorporation. Whereas most of the identified transcripts probably mimic an acceptor-stem like structure with a single unpaired residue at the 3′-end, some substrates seem to have additional unpaired nucleotides and/or rather unstable stem elements (Figure 2). This is consistent with the observation that this enzyme accepts several mini- as well as microhelix variants of tRNAs (10,11). Regarding the kinetic parameters of several selected candidate RNAs (Table 1), it is obvious that these substrates are less efficient compared to genuine tRNAs (Table 2). As *K_M_* is an indirect indication for substrate binding, it seems that the enzyme interacts with these candidates at a similar affinity as with tRNAs. The *k*_cat_ values, however, are significantly lower than those for tRNAs. This is an indication that although the artificial substrates are bound by the enzyme, catalysis per se is less efficient. A reason for this observation might be that the ‘acceptor stem’ structure of the candidates differs from genuine tRNA acceptor and TψC helices recognized by the enzyme (48,49). Hence, the 3′-hydroxyl group of the primer end is probably not optimally positioned for nucleophilic attack required for NTP incorporation. This is further corroborated by the competition experiments in Figure 3. Yeast tRNA^Phe^, representing an optimal substrate for CCA addition, is a strong competitor and increasing concentrations completely replace the candidate substrate in the reaction. The second competitor human mitochondrial tRNA^Tyr^, however, is less efficient in competition, as its structural deviations from a standard tRNA render this transcript a less accepted substrate. Here, the candidates can compete for CCA addition, indicating that these transcripts indeed represent efficient substrates for the nucleotidyltransferase.

Interestingly, 14 out of 47 analysed substrate candidates carry additional C residues fused to the incorporated CCA ends (Figure 1). This is in good agreement with a tRNA quality control system, where the addition of two or more CCA-termini serves as a degradation tag for structurally unstable tRNAs (50). As most tRNAs start with two G residues at their 5′-end, an unstable acceptor stem allows a refolding where the first CCA-end base-pairs with G1G2, mimicking a CCA-less transcript as a substrate for a second round of CCA addition. As our randomized RNA pool also starts with two consecutive G residues, it is very likely that this 5′-end combined with unstable hairpin formation allows further CCA additions, and the observed extra C and CC residues represent partial incorporations of a second CCA end. Such an addition of extra nucleotides is also observed for several of the tested candidates (Figure 2). While it is very likely that the mechanism described above is involved, it is also possible that the well-known polyC-adding function of CCA-adding enzymes (51) contributes to this substrate elongation.

### The CCA-adding enzyme prefers substrate RNAs with a 3′-terminal A residue

The majority of the identified substrate transcripts (65.9%) share an A residue located immediately upstream of the added CCA end, reflecting a strong preference of the human CCA-adding enzyme for substrates ending with this residue. This is in amazing agreement with the *in vivo* situation, where 62.2% of the tRNAs carry an adenosine residue upstream of the CCA-terminus (37). In tRNAs, this position corresponds to the discriminator base at position 73, an important identity element for many aminoacyl-tRNA synthetases (17,18,52,53). In some instances, the replacement of this nucleotide can lead to a complete identity switch of the tRNA, underscoring the importance of this identity element (35,36).

Our results show that this discriminator position is equally important for the 3′-terminal CCA incorporation. Similar to most aminoacyl-tRNA synthetases, the CCA-adding enzyme strongly prefers adenine and guanine bases as discriminators, leading to a selective and fast nucleotide addition on tRNAs with A73 in competition as well as kinetic experiments. It is very likely that the nature of the discriminator base has an impact on stability and structural organization of the tRNA 3′-end required to position the 3′-hydroxyl for nucleophilic attack at the triphosphate of the NTP to be incorporated. Depending on the discriminator base, this position can form stacking interactions with the terminal base pair 1–72 of the acceptor stem, leading to a stabilization of this helix (37,52–54). In tRNAs, the combination of a G1-C72 base pair at the end of the acceptor stem with a dangling adenosine discriminator 73 leads to the most stable structure (37,52–54). Pyrimidine discriminators, on the other hand, have much weaker stacking interactions and tend to destabilize the stem structure (37,52). The highly stable A73/G1-C72 constellation is due to a favourable stacking of the electron-rich π system of adenine on the relatively electron-poor π system of the carbonyl group-containing guanine (54). Accordingly, this constellation is found in most tRNAs (37), and a tRNA 3′-end stabilized by a stacking A73 is the preferred substrate for CCA-adding enzymes. Since these stacking interactions represent only subtle changes in the overall tRNA structure, it is very likely that binding of the tRNA substrate to the enzyme is not affected. The obtained *K_M_* values (as an indirect indication for substrate binding) for tRNA^Tyr^ transcripts with different discriminator identities support this interpretation, as they do not differ significantly (*P* = 0.3; Table 2). *k*_cat_, however, is significantly higher for tRNA^Tyr^ with A73 compared to the variants with U73 (*P* = 0.006), supporting the hypothesis that the positioning of the 3′OH group in the tRNA primer (and the subsequent nucleophilic attack on the bound NTP) is influenced by such stacking interactions.

Unfortunately, the existing co-crystal structures of class II tRNA nucleotidyltransferases do not allow a concise interpretation of the discriminator impact. While structures exist for enzymes with partial activities (CC-adding and A-adding enzymes found in some bacterial species (25,55,56)), no co-crystal structures are available for true CCA-adding enzymes. The structure of the A-adding enzyme does not allow identifying the discriminator localization, as the resolution of the tRNA 3′-end is not sufficient (9). Co-crystal structures of the CC-adding enzyme show the discriminator position in different orientations (55). The asymmetric unit contains four individual tRNA/enzyme complexes. Two complexes show an A73 discriminator stacked upon the first G–C pair of the acceptor stem. In addition, a phenylalanine residue at position 85 in the enzyme is stacked on A73, stabilizing this orientation. The complexes with the stacked discriminator are interpreted as a pre-activation state, where the tRNA primer is bound by the enzyme, but not yet positioned for nucleotide addition (55). In the two other complexes, the discriminator A73 is not stacked anymore, but is turned away from the acceptor helix. As it enters the catalytic core of the enzyme, this constellation might represent the active conformation for C-addition (55). In one of the complexes, the N6 of A73 forms a hydrogen bond to D58 in the enzyme. D58, together with D60, is known to position a Mg_2_^+^ ion required for catalysis (40). As the other bases do not carry a sterically corresponding amino group, this interaction might contribute to an A discriminator preference in CCA addition, though its actual effect remains unclear. Taken together, both A73 orientations in these complexes might contribute to the observed A73 preference in CCA addition. The stacked A73 could support to an efficient interaction between enzyme and tRNA, while N6 hydrogen bond of the unstacked A73 could lead to an optimal positioning for nucleotide addition. However, to clarify whether these A73 orientations and interactions indeed contribute to an efficient nucleotide addition, co-crystal structures of true CCA-adding enzymes with tRNAs carrying different discriminator identities are required.

The structural impact of the acceptor stem is also described for several aminoacyl-tRNA synthetases, where the discriminator does not directly interact with the synthetase but induces a conformational change of the acceptor stem that is needed for the CCA end of the tRNA to fit into the catalytic pocket of the enzyme (18,52). Another indirect effect was shown for *E. coli* tRNA^Cys^, where U73 represents an important identity element (57) that forces the formation of a tetraloop-like fold-back structure of the UCCA sequence required for efficient recognition by the cognate cysteine synthetase (58). For other aminoacyl-tRNA synthetases, however, also direct interactions via hydrogen bonding with the discriminator are described (18,54,59).

Besides aminoacylation and CCA addition, the discriminator position is also important for correct tRNA 5′-end processing in *E. coli*, where RNase P forms hydrogen bonds with the purine discriminator of pre-tRNAs (60). Furthermore, in the *tyrS* gene in *Bacillus subtilis*, the discriminator of tRNA^Tyr^ represents a recognition element for T-box regulation (61).

### tRNA^Pro^ with C73: a compromise?

The human mitochondrial tRNA^Tyr^ with an artificial cytidine discriminator represents only a rather inefficient substrate for CCA addition, which might be explained by the destabilizing effect of C73 on the acceptor stem. Surprisingly, this artificial discriminator also leads to a dramatic increase of misincorporations by the CCA-adding enzyme (Figure 6). As this tRNA starts with two G residues at the 5′-end, it is possible that the destabilizing C73 discriminator together with C74C75 of the growing CCA end induces a refolding of the acceptor stem, where some of these C residues base-pair with G1 and G2 and allow additional incorporations of CCA termini (or parts thereof). A similar refolding is probably responsible for additional C incorporations in the randomized RNA pool described above.

Yet, several tRNAs in all three kingdoms of life carry a cytidine discriminator (28,37) without erroneous CCA additions. An example is human tRNA^Pro^, starting with two G residues at the 5′-end and ending with C73, similar to the described tRNA^Tyr^ construct. Nevertheless, replacement of C73 with A73 led to an increased CCA addition, probably due to the stabilizing stacking interaction of A73. The wild-type substrate tRNA^Pro^-C73, however, was as good as a substrate as tRNA^Pro^-G73 (Figure 5B), which is in strong contrast to the situation in tRNA^Tyr^, where C73 dramatically reduced the CCA addition (Figure 5A). Again, the kinetic parameters of CCA addition to tRNA^Pro^ are in agreement with these observations. The *K_M_* values for tRNA^Pro^-C73 and tRNA^Pro^-A73 are not significantly different (*P* = 0.05), suggesting a similar tRNA binding. The *k*_cat_ values, on the other hand, show a significant 2.5-fold difference (*P* = 0.005). Obviously, the structure of tRNA^Pro^ compensates for the destabilizing C discriminator, rendering the tRNA an intermediate substrate for the CCA-adding enzyme. Comparing the secondary structures of these tRNAs, it becomes obvious that the acceptor stem of tRNA^Pro^ exhibits a high structural stability with five GC base pairs and only two AU interactions, while in tRNA^Tyr^ only two GC pairings can be observed (Supplementary Figure S4). This stable helical element might counteract the destabilizing effect of C73, leading to efficient CCA incorporation. In addition, the stable acceptor stem also reduces the error rate during CCA-synthesis, as only a small number of discriminator-dependent misincorporations were observed for this tRNA (Figure 6).

As the discriminator base is no identity element for the human prolyl-tRNA^Pro^ synthetase (ProRS) (62), the question remains as to why tRNA^Pro^ did not evolve with an adenosine discriminator instead of a cytidine. It is possible that other activities acting on this tRNA have such a preference for C73. In bacteria, tRNA^Pro^ contains a strictly conserved A73 critical for efficient aminoacylation by the bacterial ProRS (34). However, ProRS mischarges this tRNA with alanine, and the discriminator of tRNA^Pro^ is important for correct substrate selection by the bacterial trans-editing factor ProXp-ala, which hydrolyses mischarged alanyl-tRNA^Pro^ in a proof reading reaction (63–65). Preliminary work indicates that the human homologue ProXp-ala, which likewise hydrolyses alanyl-tRNA^Pro^ (66), may also recognize the discriminator base, as it preferentially deacylates human cytosolic tRNA^Pro^ over bacterial or human mitochondrial tRNA^Pro^ (K. Musier-Forsyth, personal communication). Therefore, even though human ProRS lacks specific recognition of C73 (62), the identity of this position may be conserved in higher eukaryotes to meet the substrate requirements of the proofreading enzyme, ensuring proper translation of proline codons. The change in the discriminator position of tRNA^Pro^ from A to C does not prevent CCA addition due to the stability of the tRNA^Pro^ acceptor stem, which is a conserved feature in bacteria and eukaryotes. Hence, the composition of the human tRNA^Pro^ acceptor stem reflects a compromise between the different substrate requirements of the two enzymes, ensuring efficient 3′-end maturation as well as proofreading of mischarged tRNAs. A similar compromise seems to exist for tRNAs with U73 discriminators. Besides the example of the *E. coli* tRNA^Cys^ given above, a U73-induced fold-back structure is found at the 3′-end of initiator tRNA^Met^, where it represents an important recognition element for methionine-tRNA transformylase (53).

### The class I CCA-adding enzyme also prefers an A discriminator

Similar to the human class II CCA-adding enzyme, the class I enzyme of *A. fulgidus* also shows a strong preference for tRNAs carrying an adenosine at the discriminator position (Supplementary Figure S3). While tRNA recognition involving acceptor and TψC stems is rather similar in class I and II, the nucleotide selection is quite different. Class II enzymes use a pure protein-based binding mechanism, where an arginine and an aspartic acid residue form Watson–Crick-like hydrogen bonds with ATP or CTP (40). In class I, on the other hand, a collaborative templating takes place, where the phosphate backbone of the bound tRNA together with an arginine side chain of the enzyme specifically recognizes the nucleotides to be incorporated (67,68). Hence, class I enzymes act as ribonucleoproteins (68,69). Furthermore, in class II enzymes, the tRNA rotates during nucleotide addition, while in class I no such movement was observed (55,67,68). In the kinetic analysis of the *A. fulgidus* class I enzyme, the *K_M_* values suggest that tRNA^Pro^-A73 is slightly better bound than the corresponding tRNA with C73 (Supplementary Table S2). It is possible that the formation of the ribonucleoprotein complex is more efficient with a tRNA acceptor stem carrying a stably stacked A73 position. Together with a 2.5-fold increased *k*_cat_ value for this tRNA, these data show that class I enzymes—similar to class II—prefer a tRNA with A73, indicating that primer positioning and nucleotide incorporation have similar requirements in these enzymes. Although both classes differ in several aspects in structure and nucleotide selection, Cho *et al*. suggest a rather similar mechanism of CCA addition, as both enzymes form a functionally and structurally comparable network of hydrogen bonds between NTPs and protein during catalysis (70). In the available co-crystal structures of the *A. fulgidus* enzyme in the presence of tRNA minihelices, glutamate 96 is positioned above a discriminator A73 (71) as well as G73 (72). In addition, there is a stacking interaction between both discriminators and the first G-C base pair of the corresponding acceptor stems. Hence, both purines show an identical position in the crystal, probably supporting an efficient CCA addition, as observed in the presented studies. However, no stacking of the discriminator with a phenylalanine residue is observed, as it is found in the class II enzyme. Obviously, the recognition of the tRNA primer and the discriminator position differs between class I and II, although the effect of the discriminator on CCA addition is comparable. Again, as in the case of class II enzymes, additional co-crystals with different discriminator positions are needed to clarify the discriminator effect at the structural level.

Taken together, the discriminator position that was originally defined as an identity element for aminoacyl-tRNA synthetases is equally important for other enzymes like RNase P or certain modifying enzymes (52,53,60,61,73). This group is now joined by tRNA nucleotidyltransferase that also shows a strong dependency on the nature of the discriminator base. Consequently, discriminator base identity as well as composition and/or structure of the tRNA substrates represent a compromise of the different individual enzymes acting on tRNA or tRNA precursors. As only a subset of aminoacyl-tRNA synthetases recognize the discriminator base, the high prevalence of 62.2% of adenosine at this position is obviously caused by the substrate requirements of CCA-adding enzymes and other tRNA processing activities.

## SUPPLEMENTARY DATA

Supplementary Data are available at NAR Online.

SUPPLEMENTARY DATA
