# Supplementary material for: The identity of the discriminator base has an impact on CCA addition
Source: Nucleic Acids Res. 2015 May 9;43(11):5617–29. doi: 10.1093/nar/gkv471 (PMC4477674; doi:10.1093/nar/gkv471)
Supplement: SUPPLEMENTARY DATA [file supp_43_11_5617__index.html]

The identity of the discriminator base has an impact on CCA addition — SUPPLEMENTARY DATA 

# The identity of the discriminator base has an impact on CCA addition

## SUPPLEMENTARY DATA

- SUPPLEMENTARY DATA
- SUPPLEMENTARY DATA
- SUPPLEMENTARY DATA
- SUPPLEMENTARY DATA
- SUPPLEMENTARY DATA
