# Supplementary material for: The identity of the discriminator base has an impact on CCA addition
Source: Nucleic Acids Res. 2015 May 9;43(11):5617–29. doi: 10.1093/nar/gkv471 (PMC4477674; doi:10.1093/nar/gkv471)
Supplement: SUPPLEMENTARY DATA [file supp_gkv471_nar-00733-r-2015-File008.docx]

**SUPPLEMENTARY DATA**

**Table S1: Frequencies of discriminator positions in isolated candidate substrates and naturally occurring tRNA genes.** The values for the tRNAs represent the average of discriminator identities according to Limmer et al. {Limmer 1993 #18}. In both candidates as well as tRNAs, purines are highly overrepresented at the discriminator position.

| Discriminator | Candidates | Candidates (%) | | tRNAs (%) | |
| --- | --- | --- | --- | --- | --- |
| A | 31/47 | 65.9% | 72.3% | 62.2% | 85.1% |
| G | 3/47 | 6.4% |  | 22.9% |  |
| U | 9/47 | 19.1% | 27.6% | 11% | 14.9% |
| C | 4/47 | 8.5 % |  | 3.9% |  |

**Table S2:** Kinetic parameters determined for the class I CCA-adding enzyme of *Archaeoglobus fulgidus*. Human tRNA^Pro^ with discriminator position C73 (wt) and A73 served as substrate. As this thermophilic enzyme requires an increased reaction temperature of 50°C, it was not tested with tRNA^Tyr^ variants, as the rather unstable acceptor stem of this tRNA very likely unfolds at such an increased incubation temperature.

| Substrate | *K_M_* (µM tRNA) | *k_cat_* (s^-1^) |
| --- | --- | --- |
| tRNA^Pro^-C | 1.5 +/- 0.2 | 0.014 +/- 0.001 |
| tRNA^Pro^-A | 0.6 +/- 0.2 | 0.033 +/- 0.002 |

**Figure S1.** Analysis of the randomized sequence pool. The original pool was amplified by PCR and cloned. 75 individual clones were sequenced. The 3’-terminal nucleotide is shown on the right. In 20/75 clones (27%), this position is represented by an A residue, and the other nucleotides are found at similar frequencies, showing no enrichment for A or G residues. This distribution indicates that the observed enrichment for purine ends in the substrates reflects the preference of the CCA-adding enzyme for such RNA ends.

**Figure S2.** CCA addition on candidate RNA #3. While the transcript ending with an A residue at its 3’-terminal position is readily accepted by the CCA-adding enzyme and elongated by the incorporation of CCA, a version lacking this A residue (#3 Δ3’A) is not elongated, indicating that the CCA-adding enzyme is not able to restore the missing A residue upstream of the CCA-end. This demonstrates that the observed selection of transcripts ending with A is not an artifact caused by an additional nucleotide incorporation, but reflects the substrate requirements of the CCA-adding enzyme.

**Figure S3.** The discriminator base identity has a similar effect on the CCA-addition catalysed by the corresponding enzymes of *E. coli* and *A. fulgidus*. In the time series for both enzymes, tRNA^Tyr^ with purines at position 73 is the highly preferred substrate, leading to fast and efficient CCA addition. In contrast, transcripts ending with U73 and C73 show a less efficient nucleotide incorporation. In the case of the *E. coli* enzyme, the addition of a fourth nucleotide is visible at longer incubation times. This represents a well described *in vitro* side reaction specific for the *E. coli* CCA-adding enzyme (1–3).

**Figure S4.** Secondary structure models of human tRNA^Pro^ and human mitochondrial tRNA^Tyr^ as used in this study. GC base pairs are indicated by two blue lines, AT base pairs by a single line, GU pairs by one line with a blue filled circle. Mismatches are presented as red lines. While the mitochondrial transcript shows a rather weak acceptor stem (only 2 GC base pairs), the cytosolic tRNA^Pro^ is more stable in this element. This increased stability seems to compensate for the unfavorable cytidine discriminator, allowing a rather efficient and error-free addition of the CCA terminus.

**REFERENCES**

1. Betat, H., Rammelt, C., Martin, G. and Mörl, M. (2004) Exchange of regions between bacterial poly(A) polymerase and the CCA-adding enzyme generates altered specificities. *Mol. Cell.,* **15,** 389–398.

2. Cho, H.D., Verlinde, Christophe L M J and Weiner, A.M. (2007) Reengineering CCA-adding enzymes to function as (U,G)- or dCdCdA-adding enzymes or poly(C,A) and poly(U,G) polymerases. *Proc. Natl. Acad. Sci. U S A,* **104,** 54–59.

3. Just, A., Butter, F., Trenkmann, M., Heitkam, T., Mörl, M. and Betat, H. (2008) A comparative analysis of two conserved motifs in bacterial poly(A) polymerase and CCA-adding enzyme. *Nucleic Acids Res.,* **36,** 5212–5220.
